# Sinularin Selectively Kills Breast Cancer Cells Showing G2/M Arrest, Apoptosis, and Oxidative DNA Damage

**DOI:** 10.3390/molecules23040849

**Published:** 2018-04-08

**Authors:** Hurng-Wern Huang, Jen-Yang Tang, Fu Ou-Yang, Hui-Ru Wang, Pei-Ying Guan, Chiung-Yao Huang, Chung-Yi Chen, Ming-Feng Hou, Jyh-Horng Sheu, Hsueh-Wei Chang

**Affiliations:** 1 Institute of Biomedical Science, National Sun Yat-Sen University, Kaohsiung 80424, Taiwan; sting@mail.nsysu.edu.tw (H.-W.H.); whr0319@gmail.com (H.-R.W.); rockyayaya@hotmail.com (P.-Y.G.); 2 Department of Radiation Oncology, Faculty of Medicine, College of Medicine, Kaohsiung Medical University, Kaohsiung 80708, Taiwan; reyata@kmu.edu.tw; 3 Department of Radiation Oncology, Kaohsiung Medical University Hospital, Kaohsiung 80708, Taiwan; 4 Division of Breast Surgery and Department of Surgery, Kaohsiung Medical University Hospital, Kaohsiung 80708, Taiwan; kmufrank@gmail.com (F.O.-Y.); mifeho@kmu.edu.tw (M.-F.H.); 5 Cancer Center, Kaohsiung Medical University Hospital, Kaohsiung Medical University, Kaohsiung 80708, Taiwan; 6 Department of Marine Biotechnology and Resources, National Sun Yat-sen University, Kaohsiung 80424, Taiwan; huangcy@mail.nsysu.edu.tw; 7 Department of Nutrition and Health Sciences, School of Medical and Health Sciences, Fooyin University, Kaohsiung 83102, Taiwan; xx377@fy.edu.tw; 8 Institute of Clinical Medicine, Kaohsiung Medical University, Kaohsiung 80708, Taiwan; 9 Kaohsiung Municipal Hsiao-Kang Hospital, Kaohsiung 81267, Taiwan; 10 Doctoral Degree Program in Marine Biotechnology, National Sun Yat-sen University, Kaohsiung 80424, Taiwan; 11 Department of Medical Research, China Medical University Hospital, China Medical University, Taichung 40402, Taiwan; 12 Frontier Center for Ocean Science and Technology, National Sun Yat-sen University, Kaohsiung 80424, Taiwan; 13 Department of Medical Research, Kaohsiung Medical University Hospital, Kaohsiung 80708, Taiwan; 14 Institute of Medical Science and Technology, National Sun Yat-sen University, Kaohsiung 80424, Taiwan; 15 Department of Biomedical Science and Environmental Biology, Kaohsiung Medical University, Kaohsiung 80708, Taiwan

**Keywords:** soft coral, marine natural product, oxidative stress, apoptosis, SKBR3

## Abstract

The natural compound sinularin, isolated from marine soft corals, is antiproliferative against several cancers, but its possible selective killing effect has rarely been investigated. This study investigates the selective killing potential and mechanisms of sinularin-treated breast cancer cells. In 3-(4,5-dimethylthiazol-2-yl)-5-(3-carboxymethoxyphenyl)-2-(4-sulfophenyl)-2H- tetrazolium, inner salt (MTS) assay, sinularin dose-responsively decreased the cell viability of two breast cancer (SKBR3 and MDA-MB-231) cells, but showed less effect on breast normal (M10) cells after a 24 h treatment. According to 7-aminoactinomycin D (7AAD) flow cytometry, sinularin dose-responsively induced the G2/M cycle arrest of SKBR3 cells. Sinularin dose-responsively induced apoptosis on SKBR3 cells in terms of a flow cytometry-based annexin V/7AAD assay and pancaspase activity, as well as Western blotting for cleaved forms of poly(ADP-ribose) polymerase (PARP), caspases 3, 8, and 9. These caspases and PARP activations were suppressed by *N*-acetylcysteine (NAC) pretreatment. Moreover, sinularin dose-responsively induced oxidative stress and DNA damage according to flow cytometry analyses of reactive oxygen species (ROS), mitochondrial membrane potential (MitoMP), mitochondrial superoxide, and 8-oxo-2′-deoxyguanosine (8-oxodG)). In conclusion, sinularin induces selective killing, G2/M arrest, apoptosis, and oxidative DNA damage of breast cancer cells.

## 1. Introduction

Breast cancer is the most common type of cancer to affect women. Reactive oxygen species (ROS) play an important role in breast cancer cell survival and proliferation [1], i.e., low ROS levels promote breast carcinogenesis but high ROS levels induce cell death. Moreover, cancer cells have a higher level of ROS than normal cells [2]. Since cancer cells cannot tolerate as much exogenous oxidative stress as their non-cancer counterparts, drug-induced oxidative stress can induce cancer cell death [3]. Compared to normal cells, exogenous oxidative stress easily induces ROS generation and decreases mitochondrial membrane potentials in cancer cells. These effects explain the anticancer effects of several ROS-upregulating drugs. Hence, modulating the ROS level may have potential for breast cancer therapy.

For example, 4β-hydroxywithanolide [4,5] and withanone [6] have been reported to selectively kill oral and breast cancer cells. Accordingly, ROS-upregulating drugs may differentially generate ROS to induce selective oxidative stress on cancer cells as compared to normal cells [3,7]. However, the mechanism of action of natural products in selectively killing breast cancer cells has not yet been investigated.

Effective drug development relies on identifying more and better bioactive compounds, such as lead molecules for subsequent modeling and synthesis, with selective killing effects against breast cancer cells. Some natural marine products have been found to inhibit proliferation [8,9] and induce apoptosis by modulating ROS generation [10,11,12]. Marine organisms, such as soft corals, have been found to provide highly diverse and abundant bioactive compounds against several cancer types. For example, the aquacultured soft coral *Sinularia flexibilis* (*S. flexibilis*)-derived sinulariolide has been reported to suppress the migration and invasion of bladder cancer (TSGH-8301) cells [13], and to inhibit the proliferation of oral cancer (Ca9-22) cells [14]. The wild-type soft coral (*Nephthea erecta*)-derived natural steroid 24-methyl-cholesta-5,24(28)-diene-3beta, 19-diol-7beta-monoacetate was reported to inhibit the proliferation of lung cancer H1688 cells [15]. Several novel isoprenoids isolated from the soft coral *Sarcophyton glaucum* also display cytotoxicity to several types of cancer [16].

The current study examines the marine natural product sinularin isolated from the soft coral *Sarcophyton flexibilis* [17]. The same substance has been isolated from *S. manaarensis* [18]. It is one of the main bioactive compounds in both corals, but has received little attention for its medical applications. Its anticancer effect has been demonstrated in human melanoma (A2058) cells [19] and gastric cancer (AGS) cells [20]. However, its selective killing effect on cancer was first shown in our previous study on oral cancer cells [21]. Here, we hypothesize that sinularin has selective killing potential against other types of cancer cells, such as breast cancer cells.

To test this hypothesis, we selected two types of breast cancer (SKBR3 and MDA-MB-231) cells and one type of breast normal (M10) cells to evaluate the potential selective killing effect of sinularin and to explore its antiproliferative mechanism in terms of cell viability, cell cycle distribution, apoptosis, ROS generation, mitochondrial membrane potential (MitoMP), mitochondrial superoxide, and oxidative DNA damage.

## 2. Results

### 2.1. Cell Viability of Sinularin-Treated Breast Cancer and Normal Breast Cells

**Figure 1:**
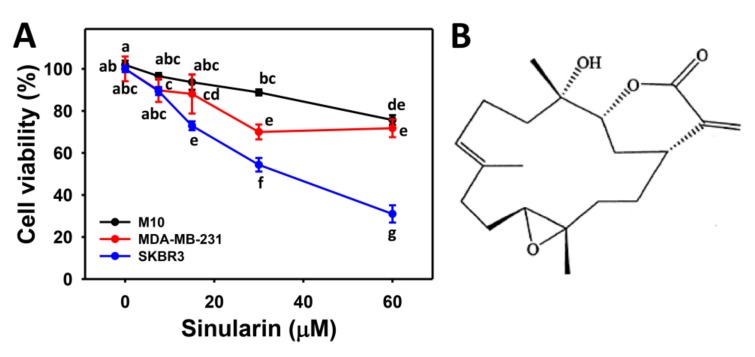
Cell viabilities of sinularin-treated breast cancer cells. (**A**) Cell viabilities. Breast cancer (SKBR3 and MDA-MB-231) cells and breast normal (M10) cells were compared. Cells were treated with 0 (DMSO only), 7.5, 15, 30, and 60 μM of sinularin for 24 h to determine cell viability by MTS assay. Data, means ± SDs (*n* = 3). Data for different treatments between different cells were compared. Treatments without the same small letters significantly differed (*p* < 0.05–0.001). (**B**) The structure of sinularin.

Figure 1 shows the cell viability (%) of two sinularin-treated breast cancer (SKBR3 and MDA-MB-231) cells with a substantial dose-responsive decrease. By contrast, the cell viability of sinularin-treated breast normal (M10) cells was only slightly decreased. Because sinularin seems to be more effective against SKBR3 (HER2+ type) than MDA-MB-231 (triple-negative type) breast cancer cells, we chose the SKBR3 cells to further examine their cytotoxic mechanisms in the following.

### 2.2. Cell Cycle Changes of Sinularin-Treated Breast Cancer Cells

**Figure 2:**
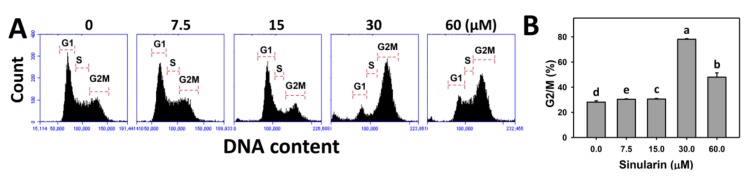
Flow cytometry cell cycle analysis of sinularin-treated breast cancer (SKBR3) cells. (**A**) Representative cell cycle patterns of sinularin-treated SKBR3 cells. Cells were treated with 0 (DMSO only), 7.5, 15, 30, and 60 μM of sinularin for 24 h. 7-Aminoactinomycin D (7AAD) was used to stain DNA content for flow cytometry. (**B**) Statistics of the percentages of cell cycle phase in Figure 2A. Data, means ± SDs (*n* = 3). Data for different treatments were compared. Treatments without the same small letters significantly differed (*p* < 0.05–0.001).

Figure 2A shows the patterns of cell cycle distribution for sinularin-treated breast cancer (SKBR3) cells. Figure 2B shows that the percentages of G2/M populations for sinularin-treated SKBR3 cells are increased as compared to the control, suggesting that sinularin arrests breast cancer cells at the G2/M phase.

### 2.3. Annexin V/7AAD-Based Apoptosis of Sinularin-Treated Breast Cancer and Normal Breast Cells

**Figure 3:**
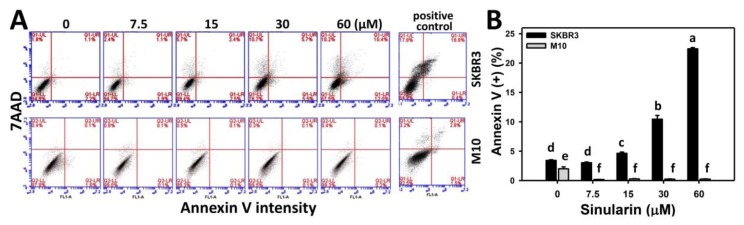
Flow cytometry of apoptosis using annexin V/7AAD changes of sinularin-treated breast cancer (SKBR3) and normal breast (M10) cells. (**A**) Representative pattern of annexin V/7AAD double staining in sinularin-treated SKBR3 and M10 cells. Cells were treated with 0 (DMSO only), 7.5, 15, 30, and 60 μM of sinularin for 24 h. Annexin V (+)/7AAD (+) and Annexin V (+)/7AAD (−) were defined as the annexin V (+) for apoptosis. Positive control treatment is 10 mM H_2_O_2_ with 10 min incubation. (**B**) Statistics of annexin V-based apoptosis for the sinularin-treated SKBR3 and M10 cells in Figure 3A. Data, means ± SDs (*n* = 3). Data for different treatments were compared. Treatments without the same small letters differed significantly (*p* < 0.05–0.001).

To examine apoptosis, the annexin V/7AAD patterns of sinularin-treated breast cancer (SKBR3) and normal breast (M10) cells were analyzed using flow cytometry. Figure 3A shows the annexin V/7AAD flow cytometric patterns for sinularin-induced apoptosis changes of SKBR3 cells (top side) and M10 cells (bottom side). Figure 3B shows that the percentages of annexin V-positive intensities for sinularin-treated SKBR3 cells increase in a dose-dependent manner at 24 h, and display higher percentages than M10 cells for all concentrations.

### 2.4. Caspase-Based Apoptosis of Sinularin-Treated Breast Cancer and Normal Breast Cells

**Figure 4:**
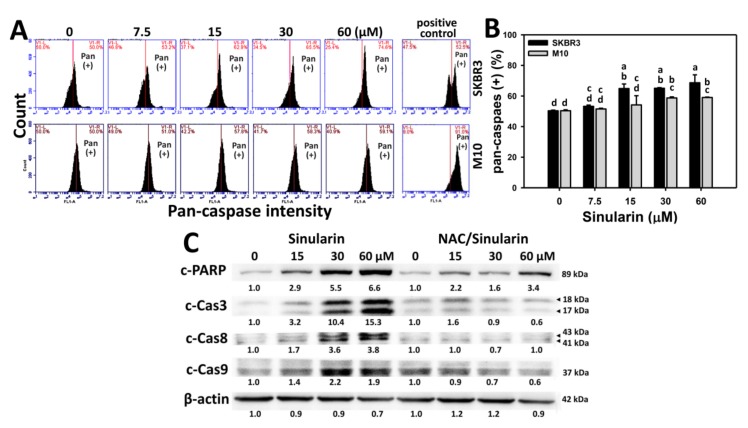
Caspase-based apoptosis assay in sinularin-treated breast cancer (SKBR3) cells. (**A**) Representative flow cytometry-based apoptosis patterns of pancaspase activity for sinularin-treated SKBR3 cells. Cells were treated with 0 (DMSO only), 7.5, 15, 30, and 60 μM of sinularin for 24 h. Positive control treatment is 10 mM H_2_O_2_ with 10 min incubation. (**B**) Statistics of pancaspase intensity positive (%) in Figure 4A. The right side labeled with Pan (+) indicates the percentage of the pancaspase-positive region in each panel. Data, means ± SDs (*n* = 3). Data for different treatments were compared. Treatments without the same small letters differed significantly (*p* < 0.05–0.001). For example, 15 μM of sinularin (a,b) in SKBR3 cells is differed significantly to 0 μM of sinularin (d) in SKBR3 cells, i.e., “a,b” without the same small letters to “d” between 15 and 0 μM of sinularin in SKBR3 cells. Similarly, 15 μM of sinularin (a,b) in SKBR3 cells is differed significantly to 15 μM of sinularin (c,d) in M10 cells without the same small letters. (**C**) Western blotting of apoptosis signaling proteins (cleaved forms of PARP (c-PARP) and caspases (c-Cas) 3, 8, and 9) in sinularin-treated SKBR3 cells. Without or with NAC (2 mM) pretreatment for 1 h, cells were respectively treated with sinularin (0, 15, 30, and 60 μM) for 24 h, i.e., sinularin or NAC/sinularin. Finally, proteins extracted from cells with sinularin or NAC/sinularin treatments were resolved in the same gel and membrane for Western blotting. β-Actin was used as an internal control.

To further examine the degree of apoptosis for sinularin-treated breast cancer (SKBR3) (top side) and normal breast (M10) (bottom side) cells, the flow cytometry-based pancaspase patterns for generic activity of caspases 1, 3, 4, 5, 6, 7, 8, and 9 [22]) are provided (Figure 4A). Figure 4B shows that the percentages of pancaspase-positive (Pan (+)) intensities for sinularin-treated SKBR3 cells are enhanced in a dose-dependent manner and display higher percentages than M10 cells ranging from 15 to 60 μM.

To further examine the detailed involvement of caspases in sinularin-induced apoptosis in breast cancer cells, Western blotting analysis for cleaved forms of poly (ADP-ribose) polymerase (PARP) and caspases 3, 8, and 9 were performed. As shown in Figure 4C (left side), cleaved forms of PARP and caspases 3, 8, and 9 were dose-responsively increased in sinularin-treated breast cancer SKBR3 cells. By contrast, the sinularin-induced cleaved form expressions of PARP and caspases 3, 8, and 9 in breast cancer (SKBR3) cells were inhibited by *N*-acetylcysteine (NAC) pretreatment (Figure 4C, right side).

### 2.5. ROS Generation of Sinularin-Treated Breast Cancer and Normal Breast Cells

**Figure 5:**
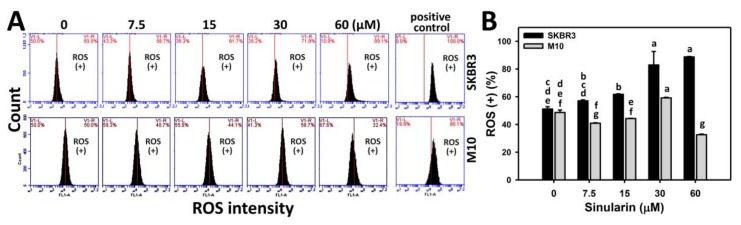
Reactive oxygen species (ROS) changes of sinularin-treated breast cancer (SKBR3) cells. Cells were treated with 0 (DMSO only), 7.5, 15, 30, and 60 μM of sinularin for 24 h. (**A**) Representative flow cytometry-based ROS patterns of sinularin-treated SKBR3 cells. The right side labeled with ROS (+) indicates the percentage of ROS-positive region in each panel. Positive control treatment is 10 mM H_2_O_2_ with 30 min incubation. (**B**) Statistics of ROS (+) intensity in Figure 5A. Data, means ± SDs (*n* = 3). Data for different treatments were compared. Treatments without the same small letters differed significantly (*p* < 0.05–0.001).

DCFH-DA-based flow cytometry was used to measure the change of sinularin (0, 7.5, 15, 30, and 60 μM)-induced ROS generation in breast cancer (SKBR3) and normal breast (M10) cells. Figure 5A shows the ROS flow cytometry patterns of SKBR3 cells (top side) and M10 cells (bottom side) after sinularin treatment for 24 h. Figure 5B shows that the relative ROS-positive staining of sinularin-treated SKBR3 cells are increased in a dose-responsive manner, and display higher percentages than M10 cells for all concentrations.

### 2.6. MitoMP of Sinularin-Treated Breast Cancer and Normal Breast Cells

**Figure 6:**
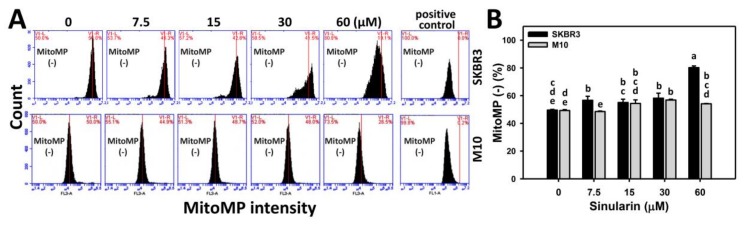
Change of MitoMP in sinularin-treated breast cancer (SKBR3) cells. (**A**) Representative dose response of MitoMP profiles for sinularin-treated SKBR3 cells using flow cytometry. Cells were treated with 0 (DMSO only), 7.5, 15, 30, and 60 μM of sinularin for 24 h. The left side labeled with MitoMP (−) indicates the percentage of the MitoMP-negative region in each panel. Positive control treatment is 50 μM carbonyl cyanide *m*-chlorophenyl hydrazone (CCCP) with 20 min incubation. (**B**) Statistics of MitoMP-negative (%) intensity in Figure 6A. Data, means ± SDs (*n* = 3). Data for different treatments were compared. Treatments without the same small letters differed significantly (*p* < 0.05–0.001).

DiOC_2_(3)-based flow cytometry was used to measure the changes of sinularin (0, 7.5, 15, 30, and 60 μM)-induced MitoMP change in breast cancer (SKBR3) and normal breast (M10) cells. Figure 6A shows the MitoMP patterns for sinularin-treated breast cancer (SKBR3) (top side) and M10 cells (bottom side) cells after 24 h. Figure 6B shows that the MitoMP-negative (%) is dose-responsively increased in sinularin-treated SKBR3 cells, and displays higher percentages than M10 cells at 7.5 and 60 μM sinularin. Consequently, the MitoMP level of SKBR3 cells was significantly decreased after sinularin treatment.

### 2.7. Superoxide Generation of Sinularin-Treated Breast Cancer and Normal Breast Cells

**Figure 7:**
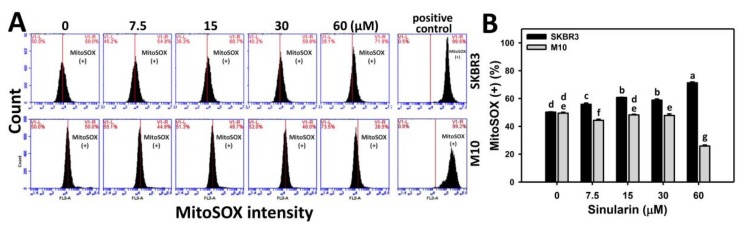
Superoxide generation of sinularin-treated breast cancer (SKBR3) cells. (**A**) SKBR3 cells treated with 0 (DMSO only), 7.5, 15, 30, and 60 μM of sinularin for 24 h were stained with MitoSOX dye. The right side labeled with MitoSOX (+) indicates the percentage of MitoSOX-positive region in each panel. Positive control treatment is 10 mM H_2_O_2_ with 30 min incubation. (**B**) Statistics of relative MitoSOX (+) fluorescent intensity (%) in Figure 7A. Data, means ± SDs (*n* = 3). Data for different treatments were compared. Treatments without the same small letters differed significantly (*p* < 0.05–0.001).

The role of oxidative stress in sinularin-treated SKBR3 and normal breast (M10) cells was examined in terms of superoxide detection. Figure 7A shows the flow cytometry-based superoxide staining (MitoSOX) patterns of sinularin-treated SKBR3 (top side) and M10 cells (bottom side) cells at 24 h incubation. Figure 7B shows that the relative MitoSOX-positive intensities (%) of sinularin-treated SKBR3 cells are dose-responsively induced, and display higher percentages than M10 cells for all concentrations.

### 2.8. Flow Cytometry-Based 8-OxodG DNA Damage Changes of Sinularin-Treated Breast Cancer and Normal Breast Cells

**Figure 8:**
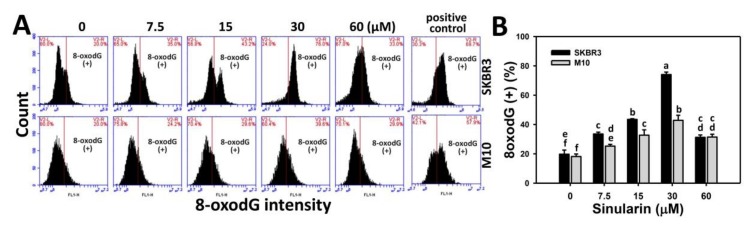
8-OxodG changes of sinularin-treated breast cancer (SKBR3) cells. (**A**) Typical 8-oxodG patterns of sinularin-treated breast cancer cells. Cells were treated with 0 (DMSO only), 7.5, 15, 30, and 60 μM of sinularin for 24 h. The right side labeled with 8-oxodG (+) indicates the percentage of 8-oxodG-positive region in each panel. Positive control treatment is 100 μM H_2_O_2_ with 24 h incubation. (**B**) Statistics of relative 8-oxodG (+) pattern (%) for Figure 8A. Data, means ± SDs (*n* = 3). Data for different treatments were compared. Treatments without the same small letters differed significantly (*p* < 0.05–0.001).

8-Oxo-2′-deoxyguanosine (8-oxodG) is the main product of oxidative DNA damage [23]. We further measured the 8-oxodG-specific expression by OxyDNA assay kit. Figure 8A shows the 8-oxodG staining-positive patterns of sinularin-treated breast cancer (SKBR3) and normal breast (M10) cells for 24 h. Figure 8B shows the 8-oxodG staining-positive expression (%) of sinularin-treated SKBR3 cells increases substantially and displays higher percentages than M10 cells ranging from 7.5 to 30 μM.

## 3. Discussion

This study investigated the modulating effects of proliferation, cell cycle progression, oxidative stress, and DNA damage in sinularin-treated breast cancer cells. The following discusses a comparison of drug sensitivity, the changes of cell cycle disturbance, possible signal transduction, and the role of oxidative stress in sinularin-treated breast cancer cells.

Different cancer cell types display different sensitivities to sinularin. For example, human melanoma (A2058) cells [19], gastric cancer (AGS) cells [20], oral cancer (Ca9-22) cells [21], and breast cancer (SKBR3) cells (the current study) respectively show IC_50_ values of sinularin with 9.28, 17.73, 23.5, and 33 μM at 24 h treatment. By contrast, sinularin is only moderately cytotoxic to normal breast (M10) cells, and shows at least 80% viability at the highest concentration (60 μM). The results suggest that sinularin has a selective killing effect against breast cancer cells, but low cytotoxicity to normal breast cells.

Several oxidative stress-inducing drugs cause G2/M arrest and delay cell proliferation [21,24,25,26,27]. Thus, it is not surprising that our results find that sinularin induces ROS generation, G2/M arrest, and apoptosis in breast cancer cells. Although, we could not confirm an increase for subG1 population, sinularin induced apoptosis in breast cancer cells as validated by annexin V/7AAD, pancaspase analyses and Western blotting. Drugs such as evodiamine [28], withametelin [29], and (−)-anonaine [30], also cause apoptosis without subG1 accumulation. Interestingly, the proportion of drug-induced apoptotic cells may change over time for subG1 accumulation [30]. For example, (−)-anonaine induced little subG1 at 24 and 48 h. However, dramatic subG1 accumulation (~40%) appeared at 72 h [30]. In any case, subG1 accumulation is not essentially related to drug-induced apoptosis. 

Moreover, we found that the levels of both intrinsic apoptotic protein c-Cas9 and extrinsic apoptotic protein c-Cas8 are increased gradually in sinularin-treated breast cancer cells. Similarly, sinularin also induces c-Cas9 and c-Cas 8 expressions in hepatocellular carcinoma cells [31] and gastric cancer cells [20]. These results suggest that the anticancer effect of sinularin is exerted by both intrinsic and extrinsic apoptosis pathways.

Sinularin was reported to induce anticancer effects against melanoma, gastric, and liver cancer cells. For example, sinularin induced apoptosis in melanoma (A2058) cells [19]. In sinularin-treated liver cancer (HepG2) cells, *Ataxia telangiectasia* mutated (ATM)/checkpoint kinase 2 (Chk2) was activated to induce DNA damages in terms of γH2AX [31]. In sinularin-treated gastric cancer (AGS and NCI-N87) cells, the phosphoinositide 3-kinase (PI3K)/AKT/mammalian target of rapamycin (mTOR) signaling was inactivated and lead to apoptosis [20]. The induction of oxidative stress was only reported in liver cancer (HepG2) cells by decreasing the MitoMP [31]. In addition, this study is the first to report the oxidative stress from mitochondrial superoxide (Figure 7) and DNA damage effect (8-oxodG) (Figure 8) of sinularin in breast cancer. The role of oxidative stress in other studies of sinularin-treated melanoma, liver, and gastric cancer cells was not investigated.

To further address the role of oxidative stress in sinularin-treated breast cancer cells, we pretreated them with the free radical scavenger NAC. We found that the sinularin-induced PARP and caspase activations were inhibited by NAC pretreatment (Figure 4C). This showed the role of oxidative stress in sinularin-induced apoptosis in breast cancer cells, consistent with our previous oral cancer cell study [21]. These results also warrant further investigation into other sinularin-induced ROS-mediated mechanisms, such as mitochondrial membrane depolarization, mitochondrial superoxide effects, and the induction of mitochondrial fission [32,33], induction of oxidative DNA damage (8-oxodG), autophagy [11,34], and endoplasmic reticulum (ER) stress [12,35].

In conclusion, the soft coral-derived natural compound sinularin induces antiproliferative and apoptotic effects on breast cancer cells and is associated with G2/M arrest and oxidative DNA damage in an oxidative stress-dependent manner. The selective killing effect of sinularin presents benefits for breast cancer therapy with few apparent side effects.

## 4. Materials and Methods

### 4.1. Cell Cultures and Drug Information

Two human breast cancer cell lines (SKBR3 and MDA-MB-231) and a normal human breast cell line (M10) were respectively ordered from the American Type Culture Collection (ATCC; Manassas, VA, USA) and Bioresource Collection and Research Center (BCRC; HsihChu, Taiwan). SKBR3 and MDA-MB-231 cells were maintained in DMEM/F12 (3:2) medium and supplemented with 10% fetal bovine serum (FBS), antibiotics, and 0.03% glutamine (Gibco, Grand Island, NY, USA). M10 cells were maintained in alpha medium with 10% FBS and regular antibiotics (Gibco, Grand Island, NY, USA). Cells were kept in a humidified atmosphere containing 5% CO_2_ at 37 °C.

Sinularin was isolated and purified from the soft corals *S. flexibilis* [17] and *S. manaarensis* [18] and dissolved in dimethyl sulfoxide (DMSO) for drug treatment. All treatments with 0 (DMSO only), 7.5, 15, 30, and 60 μM of sinularin had the same final DMSO concentration (0.2%). A free radical scavenger, NAC (Sigma; St. Louis, MO, USA) was used in a pretreatment (2 mM) for 1 h to evaluate the role played by sinularin treatment in oxidative stress [36,37,38].

### 4.2. Cell Viability

Cell viability was detected based on mitochondrial activity. We applied the 3-(4,5-dimethylthiazol-2-yl)-5-(3-carboxymethoxyphenyl)-2-(4-sulfophenyl)-2H-tetrazolium, inner salt (MTS) kit (CellTiter 96 Aqueous One Solution, Promega, Madison, WI, USA) as previously described [4]. Details of drug application are mentioned in figure legends. In brief, cells were treated with 0, 7.5, 15, 30, and 60 μM of sinularin for 24 h. MTS reagent was then added to the cells for 1 h incubation at 37 °C. The OD values at an absorbance frequency of 490 nm were measured by an ELISA Reader (EZ Read 400 Research, BioChrom Ltd., Holliston, MA, USA).

### 4.3. Cell Cycle Analysis

DNA was stained by 7AAD (Biotium, Inc., Hayward, CA, USA) for cell cycle analysis as previously described [39]. After drug treatment, cells were harvested, fixed, centrifuged, and incubated with 1 μg/mL of 7AAD in phosphate-buffered saline (PBS) for 30 min at room temperature in darkness. Finally, cell cycle analysis was performed by flow cytometry using an Accuri™ C6 (Becton-Dickinson, Mansfield, MA, USA) and its software.

### 4.4. Determination of Apoptosis by Annexin V/7AAD Assay

Annexin V (Strong Biotech Corporation, Taipei, Taiwan)/7AAD was used to detect apoptosis. After drug treatment, cells were treated with 10 μg/mL of annexin V-fluorescein isothiocyanate and 1 μg/mL of 7AAD for 30 min followed by flow cytometry using an Accuri™ C6.

### 4.5. Determination of Apoptosis by Pancaspase Activity

Caspase activity for apoptosis [40] (such as caspases 1, 3, 4, 5, 6, 7, 8, and 9 [22]) was measured by the generic caspase activity assay kit (Fluorometric–Green; ab112130) (Abcam, Cambridge, UK). After drug treatment, cells were incubated with 2 μL of 500X TF2-VAD-FMK for 2 h in a cell incubator. After washing and resuspension in an assay buffer, cells were checked for caspase activity by flow cytometry using an Accuri™ C6.

### 4.6. Determination of Apoptosis by Western Blotting

Detailed Western blotting procedures were previously described [41]. Briefly, protein lysates (30 μg) were applied for 8% sodium dodecyl sulfate polyacrylamide gel electrophoresis (SDS-PAGE), transferred to the membrane and blocked with 5% nonfat milk before antibody treatments. The information for primary antibodies (diluted 1:1000) against apoptosis are provided as follows: cleaved form of PARP (Asp214) (D64E10) XP^®^ rabbit mAb; cleaved form of caspase-8 (Asp391) (18C8) rabbit monoclonal antibody (mAb); cleaved form of caspase-9 (Asp330) (D2D4) rabbit mAb; and cleaved form of caspase-3 (Asp175) (5A1E) rabbit mAb (Cell Signaling Technology, Inc., Danvers, MA, USA). The mAb-β-actin (clone AC-15) (#A5441; Sigma-Aldrich, St. Louis, MO, USA) (diluted 1:5000) was used as an internal control. After secondary antibodies were incubated and washed, the signal was generated using ECL substrate (WesternBright™ ECL HRP: #K-12045-D50; Advansta, Menlo Park, CA, USA).

### 4.7. Determination of Intracellular ROS

ROS detecting dye, 2′,7′-dichlorodihydrofluorescein diacetate (DCFH-DA) (Sigma Chemical Co., St. Louis, MO, USA), was used to measure the intracellular ROS levels [42]. After drug treatments, cells were incubated with 10 μM DCFH-DA in phosphate-buffered saline (PBS) for 30 min at 37°C. After harvesting and resuspension in PBS, cells were studied by flow cytometry using an Accuri™ C6.

### 4.8. Determination of MitoMP

A MitoProbe^TM^ DiOC_2_(3) assay kit (Invitrogen, San Diego, CA, USA) was used to measure the mitochondrial membrane potential (MitoMP), as previously described [43]. After drug treatment, cells were washed with PBS and incubated with 10 μL of 10 μM DiOC_2_(3) in 2 mL medium/well of 6-well culture plate for 20–30 min. After washing and resuspension, cells were studied by flow cytometry using an Accuri™ C6.

### 4.9. Determination of Mitochondrial Superoxide

MitoSOX™ Red (Molecular Probes, Invitrogen, Eugene, OR, USA) reacts with mitochondrial superoxide and becomes a fluorescent molecule [44] that allows for flow cytometric quantification [14,36]. After drug treatment, cells were incubated with 5 μM MitoSOX at 37°C for 30 min. After harvesting and resuspension in PBS, cells were studied by flow cytometry using an Accuri™ C6.

### 4.10. Determination of 8-OxodG

A fluorometric OxyDNA assay kit (#500095; EMD Millipore, Darmstadt, Germany) was used to measure 8-oxodG levels for flow cytometry analysis [45,46]. After drug treatment, cells were fixed, washed, centrifuged, and resuspended in 1 mL of kit-provided washing solution. After harvesting, cells were incubated with 100 μL of 10% dye in a washing solution for 1 h. Finally, 900 μL of PBS were added to the cell resuspension for flow cytometry (Accuri™ C6).

### 4.11. Statistical Analysis

Data are presented as mean ± SD. Group differences were determined by JMP^®^ 12 software (SAS Institute, Cary, NC, USA) with one-way analysis of variance (ANOVA) and the Tukey HSD post hoc test. Treatments without the same small letters differed significantly.
