# Correction: Huang, H.W.; et al. Sinularin Selectively Kills Breast Cancer Cells Showing G2/M Arrest, Apoptosis, and Oxidative DNA Damage. *Molecules* 2018, *23*, 849

**DOI:** 10.3390/molecules23071670

**Published:** 2018-07-09

**Authors:** Hurng-Wern Huang, Jen-Yang Tang, Fu Ou-Yang, Hui-Ru Wang, Pei-Ying Guan, Chiung-Yao Huang, Chung-Yi Chen, Ming-Feng Hou, Jyh-Horng Sheu, Hsueh-Wei Chang

**Affiliations:** 1 Institute of Biomedical Science, National Sun Yat-Sen University, Kaohsiung 80424, Taiwan; sting@mail.nsysu.edu.tw (H.-W.H.); whr0319@gmail.com (H.-R.W.); rockyayaya@hotmail.com (P.-Y.G.); 2 Department of Radiation Oncology, Faculty of Medicine, College of Medicine, Kaohsiung Medical University, Kaohsiung 80708, Taiwan; reyata@kmu.edu.tw; 3 Department of Radiation Oncology, Kaohsiung Medical University Hospital, Kaohsiung 80708, Taiwan; 4 Division of Breast Surgery and Department of Surgery, Kaohsiung Medical University Hospital, Kaohsiung 80708, Taiwan; kmufrank@gmail.com (F.O.-Y.); mifeho@kmu.edu.tw (M.-F.H.); 5 Cancer Center, Kaohsiung Medical University Hospital, Kaohsiung Medical University, Kaohsiung 80708, Taiwan; 6 Department of Marine Biotechnology and Resources, National Sun Yat-sen University, Kaohsiung 80424, Taiwan; huangcy@mail.nsysu.edu.tw; 7 Department of Nutrition and Health Sciences, School of Medical and Health Sciences, Fooyin University, Kaohsiung 83102, Taiwan; xx377@fy.edu.tw; 8 Institute of Clinical Medicine, Kaohsiung Medical University, Kaohsiung 80708, Taiwan; 9 Kaohsiung Municipal Hsiao-Kang Hospital, Kaohsiung 81267, Taiwan; 10 Doctoral Degree Program in Marine Biotechnology, National Sun Yat-sen University, Kaohsiung 80424, Taiwan; 11 Department of Medical Research, China Medical University Hospital, China Medical University, Taichung 40402, Taiwan; 12 Frontier Center for Ocean Science and Technology, National Sun Yat-sen University, Kaohsiung 80424, Taiwan; 13 Department of Medical Research, Kaohsiung Medical University Hospital, Kaohsiung 80708, Taiwan; 14 Institute of Medical Science and Technology, National Sun Yat-sen University, Kaohsiung 80424, Taiwan; 15 Department of Biomedical Science and Environmental Biology, Kaohsiung Medical University, Kaohsiung 80708, Taiwan

The authors wish to make the following correction to their paper [1]. We found that there is an error at the bottom of Figure 6A (MitoMP), which is the same as the bottom of Figure 7A (MitoSox). As the names MitoMP and MitoSox are similar, we made this mistake during revisions of the manuscript. The corrected Figure 6A is as follows:

The authors would like to apologize for any inconvenience caused to the readers by these changes which do not affect the scientific results. The manuscript will be updated and the original will remain on the article webpage, with a reference to this Correction.

## Figures and Tables

**Figure 6:**
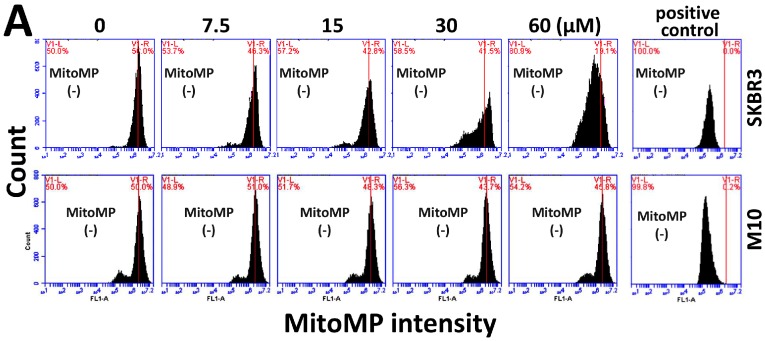
Change of MitoMP in sinularin-treated breast cancer (SKBR3) cells. (**A**) Representative dose response of MitoMP profiles for sinularin-treated SKBR3 cells using flow cytometry. Cells were treated with 0 (DMSO only), 7.5, 15, 30, and 60 μM of sinularin for 24 h. The left side labeled with MitoMP (−) indicates the percentage of the MitoMP-negative region in each panel. Positive control treatment is 50 μM carbonyl cyanide m-chlorophenyl hydrazone (CCCP) with 20 min incubation.
